# Fabrication of ECM Mimicking Bioactive Scaffold: A Regenerative Approach for MSC Mediated Applications

**DOI:** 10.1155/2023/6282987

**Published:** 2023-05-20

**Authors:** Pavani Koka, Yamini Chandramohan, Elumalai Perumal, Avinash Kavarthapu, Anuradha Dhanasekaran, Anusha Chandran, Krishnamoorthy Gunasekaran

**Affiliations:** ^1^Centre for Biotechnology, Anna University, Chennai, 600 025 Tamil Nadu, India; ^2^Bioscreen Instrumentation Pvt Ltd, Ashok Nagar, Chennai, Tamil Nadu, India; ^3^Center for Transdisciplinary Research, Department of Pharmacology, Saveetha Dental College, Saveetha Institute of Medical and Technical Sciences, Chennai, Tamil Nadu, India; ^4^Department of Periodontology, Saveetha Dental College, Saveetha Institute of Medical and Technical Sciences, Chennai, Tamil Nadu, India; ^5^Cancer and Stem Cell Biology Laboratory, Centre for Biotechnology, Anna University, Chennai, 600 025 Tamil Nadu, India; ^6^Department of Biotechnology, Karpagam Academy of Higher Education, Coimbatore, Tamil Nadu, India; ^7^Department of Medical Biochemistry, College of Health Sciences, Dambi Dollo University, P.O. Box 360, Kelam Welega Zone, Oromia Region, Ethiopia

## Abstract

Biomaterials are feasible resources that aids to replace damaged structures in our bodies. The most biologically active flora is *Aloe vera* which has many bioactive compounds that are anti-inflammatory, antimicrobial, and have ECM mimicking protein content which helps in the healing of wounds and also acts as an ECM factor for stem cell homing and differentiation. The *Aloe vera* containing 10 *w*/*v* of gelatin was lyophilized. Scaffolds had sharper morphology, greater hydrophilic properties, and a Young's modulus of 6.28 MPa and 15.9 MPa of higher tensile strength are desirable. In tissue engineering and regenerative medicine, biologically active scaffolds have been producing hopeful outcomes in both restoration and replacement, respectively. The objective of the present investigation is to test the idea that incorporating gelatin to *Aloe vera* scaffolds might enhance their structure, good biocompatibility, and possibly even bioactivity. The SEM picture of the composite scaffold revealed pore walls. The scaffolds had linked pores with diameters ranging from 93 to 296 *μ*m. *Aloe vera* and the matrix interact well, according to the FTIR study, which could lead to a reduction in the amount of water-binding sites and a reduction in the material's ability to absorb water. *Aloe vera* with 10% gelatin (AV/G) scaffold was investigated for different biological reactions of human gingival tissue mesenchymal stem cells (MSCs) in terms of cell proliferation, morphology, and cell migration. The results demonstrated the potential of the AV/G scaffold as a biomaterial that offers new insight in the field of tissue engineering.

## 1. Introduction

The field of regenerative medicine encompasses tissue engineering and stem cell technology. Organ regeneration is significantly impacted by the biomedical-related research areas of tissue engineering and regenerative medicine (TERM). It incorporates clinical research along with concepts from fields like biotechnology, mechanics, cell biology, and materials science. By constructing an environment that promotes cell growth and proliferation, tissue engineering is aimed at replacing and repairing damaged tissues [1] and generation of new tissue or organ which stimulation of the extracellular matrix [1, 2]. Scaffolds are made to encourage cell-biomaterial interaction, cell attachment, proliferation, and motility; and also make it easier to transfer mass, nutrition, and regulatory factors so that cells can survive, proliferate, and differentiate; further, allow for controlled degradation that simulates the pace of tissue regeneration under the culture conditions of interest; and have acceptable mechanical characteristics like tensile strength, porosity, and elasticity which are required to provide temporary structural strength and better spatial reorganization of the tissue until the formation of complete tissue to provoke a low level of toxicity or inflammation in the animal model [3]. Unlike simple injection of cells, scaffolds have the benefit of limiting cell loss and enabling cell transfer into a defective spot [4]. Natural biomaterials can be fabricated to offer a similar biological environment mimicking extracellular matrix (ECM) to provide better interaction with cells and for replacement of normal tissue without inducing inflammation. Scaffolds made with natural biomaterials have bioactive compounds which also help in efficient treatment and bioengineering for organ replacement. Biomaterials replicate physicochemical and biological properties and provide native ECM tissue replacement [5].

According to the World Health Organization (WHO), the most physiologically active flora is *Aloe vera* from the *Liliaceae* family [6]. There are 75 components that are bioactive in it. The glycoproteins accelerate cell proliferation to aid in the healing of wounds. It also has aloe-emodin which has antiviral, antibacterial, and anticancer properties [7]. Monosaccharides found in it are said to have excellent anti-inflammatory, anticancer, antiallergic, antimicrobial, and antitumor capabilities. Vital minerals like calcium, chromium, copper, iron, selenium, magnesium, manganese, phosphorus, chlorine, potassium, sodium, and zinc are required for metabolism and are crucial for the enzymes' proper operation [7]. There are sixteen different enzymes found in *Aloe vera*, which are frequently used as antibacterial, antifungal, antiviral, anti-inflammatory, and analgesic agents (bradykinase) for purposes [7]. Topographically, it has been observed that characteristics including holes, ridges, groves, fibres, nodes, or a mix of these can affect how cells behave [8]. Gelatin is a hydrolyzed version of collagen. Bovine and porcine skin serves as the primary source of collagen and gelatin [9]. Gelatin may form polyion complexes and is a biodegradable and biocompatible polymer. Gelatin is frequently utilised for medication and cell delivery in tissue engineering applications to target many tissues, including bone, cartilage, and skin, as a result of these qualities [10, 11].

The ability to self-renew, proliferate, and differentiate into multiple cell lineages are all characteristics of stem cells. Mesenchymal stem cells (MSCs) have demonstrated considerable potential in numerous animal studies and clinical trials as regenerative medicine. Signaling molecules, cell-to-cell contact, and stem cells' interactions with their surrounding cells to form an extracellular matrix (ECM) make up a niche [12]. Biomaterials, which act as artificial ECM, mimic the biological and mechanical properties of the native ECM found in tissues throughout the body [13]. Scaffolds with bioactive compounds can home a microenvironment that defines the fate of the cells. Stem cells can be appropriately differentiated depending on the niche when implanted with scaffold at a specific site for the reconstruction of specific tissues. In our current study, we have fabricated *Aloe vera*/gelatin (AV/10% gel) scaffold. The scaffold was further characterized for its physicochemical properties. Ethical clearance was obtained from the Department of Periodontology at Saveetha Dental College (SRB/SDMS15PER1). Stem cells from the patient's human gingival tissue were collected, isolated, and characterized using flow cytometry and confocal studies. Alkaline phosphatase and oil red O staining were used to confirm the osteogenic and adipogenic differentiation of HG Stem cells. We have employed Stem cells on a scaffold to check biocompatability. However, reconstruction in combination with stem cell and tissue engineering approaches will be a new insight to be used for tissue repair and restoration, and the ultimate outcomes will be patient safety and satisfaction.

## 2. Materials and Methods

Aloe barbadensis (*Aloe vera*) from the botanical garden, we bought gelatin from bovine skin from Sigma (USA), (Type B, powder, Bioreagent, appropriate for cell culture), isopropyl alcohol (IPA: C3H8O), ethanol (Ethyl Alcohol: C2H6O) from the Hayman, Dulbecco's Modified Eagle's medium-F12 (DMEM-F12), 1X Phosphate Buffer Solution (PBS), carbinol, chloroform, FBS (fetal bovine serum) GIBCO, trypsin, dexamethasone (Sigma), 3-isobutyl-1-methyl-xanthine (IBMX, Sigma), insulin-transferrin-selenium (ITS), indomethacin (Sigma), ascorbate-2 phosphate, *β*-glycero phosphate (Sigma), and MTT [3-(4, 5-dimethyl-thiazol)-2, 5-di-phenyl tetrazolium bromide] was purchased from Invitrogen. 4% paraformaldehyde (R&D Systems), F-actin conjugated phalloidin (red) (Invitrogen), DAPI (Sigma), CD34, CD45, CD73, CD90, CD105, and IgG anti-mouse-PE-tagged secondary antibody from the Immuno Tools, Friesoythe, Germany.

### 2.1. Scaffold Preparation

The *Aloe vera* leaf was cleaned and wiped with isopropyl alcohol. Then, the spines and outer layer of the *Aloe vera* leaf were removed. The *Aloe vera* gel was extracted from the *Aloe vera* leaf. Then, the aloe gel was blended with ethanol (for 20 ml of *Aloe vera* gel, 2 ml of ethanol). Then, a 10% *w*/*v* of gelatin was added to *Aloe vera* to make the gel. The gelatin and *Aloe vera* gel were mixed using a magnetic stirrer at 40°C. The gel was poured into Petri plates and stored at -80°C.

### 2.2. Lyophilization (Freeze Drying)

The *Aloe vera*/gelatin in Petri plates were placed at -80° C overnight before freeze-drying. Then, the Petri plates were placed in the lyophilizer at -143°C for 4 hours. After freeze-drying scaffolds, were obtained.

### 2.3. Mesenchymal Stem Cell Isolation from Human Gingival Tissue

Gingival tissue was obtained either during the crown lengthening procedure or an operculectomy in patients who fall in the age group of 15-35 years old of either gender. The site where sampling was obtained was assessed before sampling to check for any clinical signs of inflammation. Any supragingival plaque which was present was removed by thorough scaling. Local anaesthesia containing 2% lidocaine with 1: 80,000 concentration of adrenaline was given to the site from where the sample was obtained. Using a scalpel number 12 or 15 was used to excise the tissue from the operculectomy site and for crown lengthening, respectively. The obtained tissue was rinsed with saline to remove any blood clots present in the tissue. Any loose fibrin tags were removed with either a scalpel or scissors. The tissue was then immediately placed in a solution containing phosphate buffer saline (PBS) and an antibiotic containing 100 U ml^−1^ penicillin, 100 *μ*g/ml, streptomycin, 100 *μ*g/ml amphotericin B, with a ratio of 1 : 1. The medium is then transported to the laboratory for further culturing.

After obtaining the tissue, the tissue was placed in a Petri plate with sterile PBS solution (without Ca and Mg) along with an antibiotic and antimycotic solution containing 100 U/ml penicillin, 100 *μ*g/ml, streptomycin, 100 *μ*g/ml, and amphotericin B. The tissue was washed thrice with 1X PBS to remove any debris or blood clots present on the tissue surface. It was then gently scraped off with a surgical blade to remove any dead cells and minced into 1 mm pieces with the help of a surgical blade and forceps. Tissues were re-suspended and incubated in 0.3 U of collagenase type IV enzyme for 40 minutes in a CO_2_ incubator at 5% CO_2_, 95% humidity, and 37°C. After the incubation period, it was centrifuged at 1000 rpm at 4°C for 10 minutes. The supernatant was discarded, and the pellet was resuspended with 1 ml of 20% serum DMEM: F12 media.

### 2.4. Characterization of Mesenchymal Stem Cells

- Adipogenic differentiation: cells were grown in DMEM/F12 media with 10% serum along with the following reagents for adipogenic differentiation: 1 *μ*M dexamethasone, 500 *μ*M 3-isobutyl-1-methyl-xanthine (IBMX), insulin-transferrin-selenium (ITS), 60 *μ*M indomethacin, and 5 g/ml insulin as a supplemental factor. On the day 14, the intracellular buildup of lipid-rich vacuoles stained with oil red O revealed adipogenic differentiation. Cells were fixed with 4% paraformaldehyde for 30 minutes, washed, and stained for 20 minutes with a working solution of 0.16% oil red O
- Osteogenic differentiation: the cells were given 10% serum DMEM/F12 medium with 50 *μ*M ascorbate-2 phosphate, 10 mM glycerophosphate (Sigma), and 0.1 *μ*M dexamethasone added as supplements for 14 days. The formation of mineralized calcium phosphate as determined by the ALP stain served as evidence of osteogenesis. Cells were stained with 10% NBF and DMF for 60 mins at room temperature after being fixed with 4% paraformaldehyde
- Flow cytometer: we used flow cytometry to assess the expression of cell surface proteins in the cells. The software used for analysis was Kaluza Flow Analysis Software, and the flow cytometry was from a Beckman Coulter FC500. A maximal concentration of antibodies was used to stain the cells after they had been pelleted and reconstituted in PBS at a concentration of 1 × 10^5^ cells/*μ*l. For 45 minutes, cells were incubated at room temperature in the dark. Following incubation, cells were resuspended in 0.25 ml of cold PBS after being washed three times with PBS. The following primary antibodies were incubated with the MSCs: CD34, CD45, CD73, CD90, and CD105 (Immuno Tools). The antibodies have been raised against humans. Markers that were not conjugated were treated with an anti-mouse PE secondary antibody (Immuno tools). Unstained cells were used to set up the flow cytometer. To remove junk, forward scatter was used to gate cells. We eliminated the contribution of unstained cells in the measurement channel to get rid of any potential auto-fluorescence of MSCs. For each analysis, a minimum of 10,000 events were counted

### 2.5. Physical Characterization of Scaffold

#### 2.5.1. Scanning Electron Microscopy (SEM)

Electron microscopy is a precise tool that can precisely photograph bio-nanostructures, monitor biomaterials within nanomaterials, assess physical attributes, and even ascertain composition. SEM micrographs are helpful for analysing the structure of the surface because they produce three-dimensional images of a composite scaffold when they photograph a surface with an electron beam. Porosity-containing pore networks tying scaffold interiors to surface holes were observed using SEM images.

#### 2.5.2. Fourier Transform-Infrared Spectroscopy (FTIR)

FTIR is a method of analysis used to distinguish between organic and, in certain situations, inorganic materials. This method plots the wavelength vs. the amount of infrared radiation that the sample material absorbs. Molecular parts and structures are recognized through infrared absorption bands. Plots of intensity vs. wavenumber are typically used to illustrate FTIR spectra (in cm^−1^). The wavelength's reciprocal, wavenumber, is the wavelength. Plotting the intensity as a percentage of light transmittance or absorption at each wavenumber is possible.

#### 2.5.3. Differential Scanning Calorimeter (DSC)

A thermal analysis tool called a DSC measures temperature and heat flow in relation to material transitions as a function of temperature and time. It is used to measure a number of the composite scaffold's distinguishing characteristics. This method allows for the observation of fusion, crystallisation, and glass transition temperatures (Tg). It utilised to examine the chemical reactions and stability of oxidation in the samples and utilised for researching liquid crystals. The modest energy changes that take place as matter transforms from a solid to liquid crystals and from a liquid crystal to an isotropic liquid can be seen via DSC.

#### 2.5.4. X-Ray Diffraction (XRD)

Using an X-ray diffractometer (Rigaku, model Mini flex600, Japan), with a step angle of 2°C/min, X-ray patterns of the gelatin-based scaffold were recorded over a range of 5–60°.

#### 2.5.5. Contact Angle

The contact angle is the angle traditionally measured through the liquid when a liquid-vapour contact meets a solid surface. There is a specific equilibrium contact angle for every system comprising a given solid, liquid, and vapour at a given temperature and pressure. It measures the wettability of a solid surface area.

#### 2.5.6. Tensile Strength

The tensile test is one of the easiest and most commonly performed mechanical tests. Material characteristics can be ascertained by measuring the amount of force needed to stretch a specimen to its breaking point. The maximum stress that a composite structure can bear while being stretched or pulled before breaking is how ultimate tensile strength is calculated.

#### 2.5.7. Confocal Microscopy

Confocal microscopy has shown advantages for visualization of the external and internal surfaces of scaffold. Pore size and shape varied between random and aligned scaffolds. Fiber packing density, fiber alignment, and the three-dimensional view can also be analyzed. *Aloe vera* has an autofluorescence (green).

### 2.6. Biological Characterization of Scaffold

#### 2.6.1. MTT Assay

5 × 10^3^ cells/well were plated on the scaffold in a 96-well plate. After 24 hours, a MTT assay was performed. Another plate was also seeded and incubated for 72 hours. On the day of the MTT assay, media was removed and 100 *μ*l of 1X PBS and 10 *μ*l of MTT were added to each well. Incubate it for 3 hours in the dark at 37°C incubator. After the incubation, MTT and PBS were removed, and 100 *μ*l of DMSO was provided with each well, followed by a 15-minute dark incubation. This was read at 492 nm and 650 nm in the Tecan Spark multimode reader, and the graph was plotted.

#### 2.6.2. Swelling Ratio

The swelling capacity of a scaffold is determined by the amount of liquid that can be absorbed. Swelling ratio formula

(1) $$\begin{array}{c} G=\frac{\left(Wt–Wo\right)}{Wo\times 100}. \end{array}$$

*Wt* is the weight of the wet scaffold; *Wo* is the weight of the initial scaffold.

The absorption capacity of the scaffold was determined using distilled water, Dulbecco's modified Eagle's medium, and phosphate buffer saline at 37°C, pH 7.4.

#### 2.6.3. Analysis of Amino Acids Using Reverse-Phase HPLC

A reverse-phase liquid chromatographic method for the separation and relative quantification of PITC-derivatized amino acids was carried out based on the method described by Hariharan et al. [14]. Briefly, 100 *μ*L of *Aloe vera* gel was deproteinized by mixing with acetonitrile (lysate:acetonitrile (60 : 40)), vortexed, and centrifuged for 1 min at 9000 rpm. After vacuum centrifugal drying of the 100 *μ*l of the supernatant, 10 *μ*l of methanol were added. Vacuum drying is followed by combining a solution of water : triethylamine (2 : 1 : 1 *v*/*v*). The samples that had been vacuum evaporated were treated with 20 *μ*l of ethanol, water, triethylamine, and PITC (7 : 1 : 1:1 *v*/*v*), mixed, and incubated for 10 minutes before being vacuum dried. Prior to HPLC injection, the samples were mixed in 750 *μ*l of sodium acetate (pH 7.5)-acetonitrile (98 : 2 *v*/*v*) solution and filtered with 0.2 *μ*m filter. An Agilent 1200 infinite HPLC system with autosample injection was used for HPLC analysis. A C18 column (150 × 4.6 mm I.D., 3 m) (Inertsil-ODS-2) thermostated at 41°C and a straightforward multistep linear gradient of two solvents were used for the HPLC separation. Water-acetonitrile (40 : 60 *v*/*v*) was solvent B; 0.05 M sodium acetate (pH 5.1)-acetonitrile (98 : 2 *v*/*v*) served as solvent A. A UV detector set to 254 nm was used to detect the separated PITC amino acid derivatives using a 20 *μ*l injection volume. To verify the process, a standard run using the amino acid standard (Sigma) was carried out. The relative quantification analysis was performed utilising the known quantity of norleucine and the area under curve (AUC) values of the corresponding amino acid peaks.

#### 2.6.4. Confocal Studies

2 × 10^4^ MSCs were seeded onto the *Aloe vera*/10% gelatin scaffold in a 12-well plate and incubated for 24 hours. The cells were washed three times with 1X PBS after the medium was removed. For 20 minutes at 4°C, 4% paraformaldehyde was used to fix the cells to the scaffold. Paraformaldehyde was removed, and the cells were rinsed thrice with 1X PBS. DAPI and F-actin-conjugated phalloidin (red) were used to stain the cells (blue). The cells on the scaffold were viewed under confocal microscopy with muted green fluorescence to avoid background disturbance.

#### 2.6.5. Scratch Assay

A scratch assay was performed to analyze the wound healing property. 2 × 10^5^ cells/well of human gingival stem cell-like cells were seeded onto six-well culture plates in a CO_2_ incubator at 5% CO_2_ and 95% humidity at 37°C. Cells were left for 80% confluency. The monolayer of MSCs was scratched using a 200 *μ*l tip to create a wound. The detached cells were removed by washing with 1X PBS and 20% serum DMEM: F12 media with AV/G for 48 h, along with a control group for 48 h. After incubation, the wells were washed and fixed in 4% paraformaldehyde. Photographs were taken using an inverted microscope (Euromex, The Netherlands).

### 2.7. Statistical Analysis

All the results were shown as the mean ± SD. A statistically significant difference was determined by the student's *t* test. A *p* value < 0.05 was regarded as statistically significant.

## 3. Results

Reconnoitering cutting-edge technology solutions to enhance healthcare for a diseased population, still remains to be a global challenge. One innovative idea among several that have been developed into a promising strategy to fulfill patients' future requirements is tissue engineering and regenerative medicine (TERM). It is now possible thanks to the development of tissue engineering and regenerative medicine (TERM). By combining cells, scaffolds, and growth factors, tissue engineering can restore or replace damaged tissues. Although regenerative medicine integrates immunomodulation, gene therapy, and cell-based therapy to promote in vivo organ regeneration, TERM is aimed at establishing three-dimensional (3D) cells or organoids in a biomaterial scaffold structure, which mimics a structural and functionality of a live tissue or organ that can be used to regenerate and repair damaged tissue or organs. The bioscaffold must be able to sustain gaseous exchange, the movement of nutrients and waste, and cell growth as a minimum need.

### 3.1. Scaffold Fabrication and Isolation and Differentiation of Human Gingival Tissue Stem Cells

**Figure 1:**
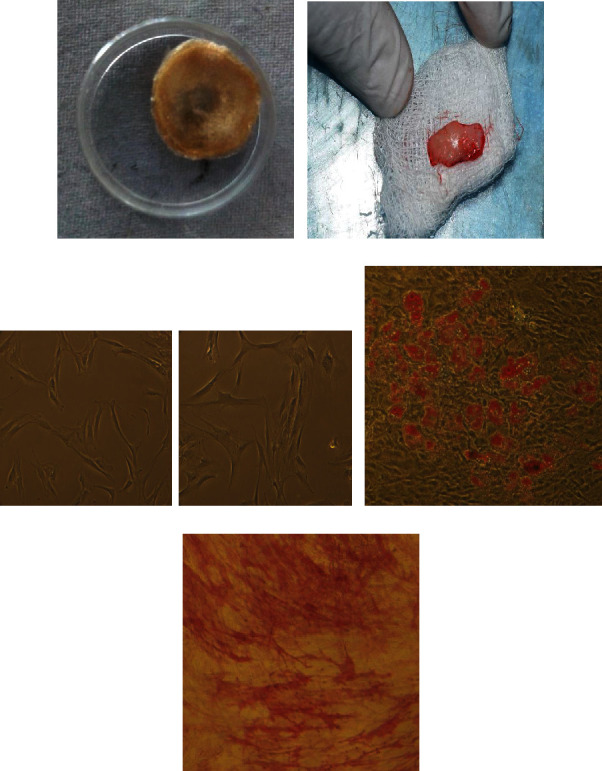
(a) *Aloe vera* with 10% gelatin (AV/G) freeze-dried scaffold. (b) Gingival tissue collected from human dental tooth pulp. (c) Phase contrast images of MSCs at 10× magnification. Human mesenchymal stem cells isolated from gingival tissue. (d) Red oil droplets represent adipogenic differentiation by oil red O staining. (e) Pink colour stain shows phosphate deposition in the nucleus, representing osteogenic differentiation by an alkaline phosphatase.

**Figure 2:**
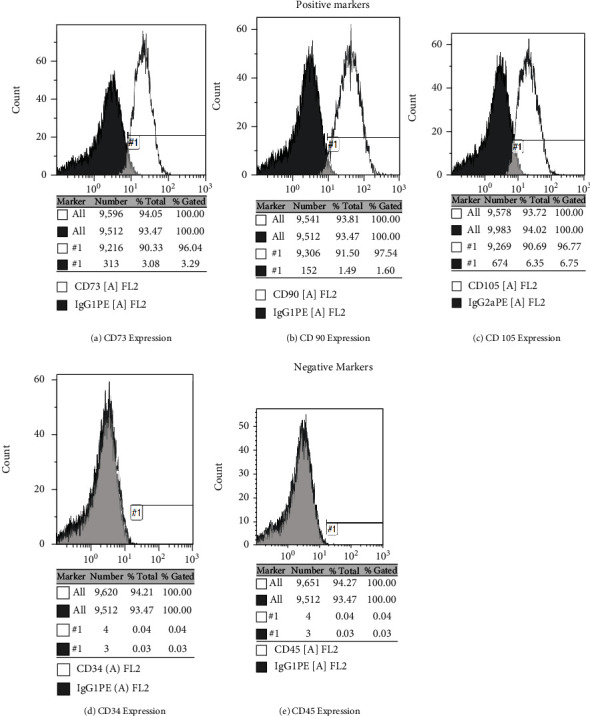
Histograms representing flow cytometry analysis. (a) 96.04% of CD73 MSC positive marker expression. (b) 97.54% of CD90 MSC-positive marker expression. (c) 96.77% of CD105 MSC positive marker expression. (d) 0.04% of CD34 MSC negative marker expression. (e) 0.04% of CD73 MSC negative marker expression.

*Aloe vera* gelatin scaffold was fabricated at a 10% concentration of gelatin and further characterized physicochemically.  Figure 1 shows fabricated AV/G (*Aloe vera*/10% gelatin) scaffold. Human gingival tissue (Figure 1(b)) from patient dental samples is collected from Saveetha Dental College. Gingival tissue was processed, and stem cell-like cells were obtained from 14–21 days of culture in DMEM: F12 with 20% FBS. Spindle-shaped, elongated stem cell-like cells outgrow from the Human gingival tissue was observed in Figures 1(a) and 1(b)). Further, to characterize these cells to be stem cells, differentiation ability of the cells was analyzed.  Figure 1(d) shows the differentiation of human gingival stem cells to adipocytes and analyzed using oil red O staining.  Figure 1(e) shows the differentiation of human gingival stem cells to osteocytes using alkaline phosphatase staining. These stem cell-like cells were further characterized with the expression of positive markers: CD73/CD90/CD105 and nonexpressed negative markers: CD34/CD45 using flow cytometer analysis. The isolated cells observed have much significant level of 94.05% CD73-positive cells (Figure 2), 93.81% of cells positive for CD90 and CD105 93.72% cells positive shown stem cells positive. There is not much significant level of negative marker CD34/CD45 expression.

### 3.2. Physical Characterization of Scaffold

**Figure 3:**
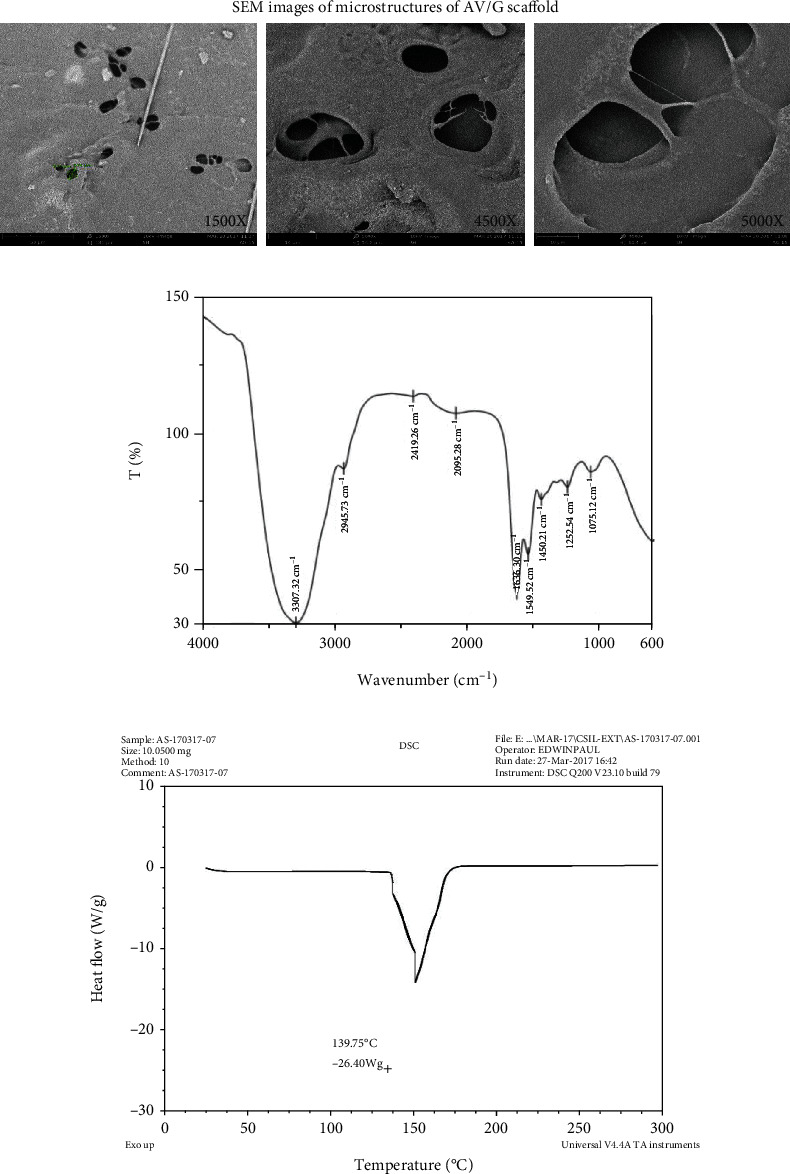
SEM images of the microstructures of the AV/G scaffold which shows the pore distribution of the cross section of the scaffold. (a) Represents 1500×, 4500×, and 5000× magnifications of SEM images of AV/G scaffold. (b) FTIR spectra of an AV/G scaffold. (c) Differential scanning calorimetric analysis of AV/G scaffold.

SEM images of the AV/G scaffold revealed that the AV/G scaffold was found to have a porous surface with random orientation. The SEM images (Figure 3(a)) of the freeze-dried AV/G scaffold showed minimal porosity at 1500×, 4500×, and 5000× magnifications. FTIR (Fourier transform infrared spectroscopy) has been used to validate and define the components in a biological composite system containing two or more components of the biomaterials *Aloe vera* and gelatin.  Figure 3(b) reveals typical band assignments for gelatin where amide A (N-H stretching vibration) was observed at 3300 cm^*-*1^, and the band absorbed by *Aloe vera* was 3314 cm*^−1^.* Further, amide I was expressed at the range of 1633–1639 cm^−1^, amide II was observed at the range of 1546–1551 cm^−1^, and amide III was observed in the range of 1239–1241 cm^−1^. The absorption peak observed around 1450 cm^−1^ for carboxyl –COOH stretch bands and C-O stretching of polysaccharides of *Aloe vera* was observed at 1079 cm^−1^.

**Figure 4:**
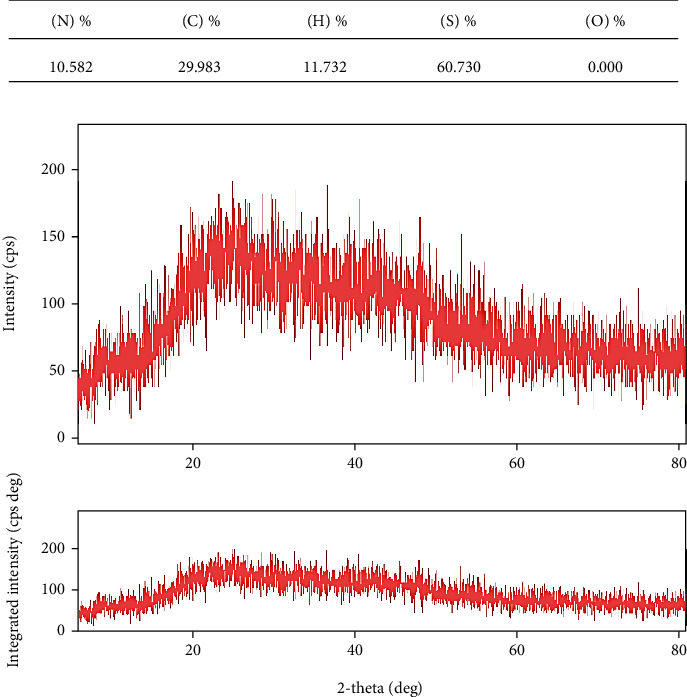
(a) Shows CHNS estimation of the AV/G scaffold. (b) Represents X-ray diffraction studies of the AV/G scaffold.

**Figure 5:**
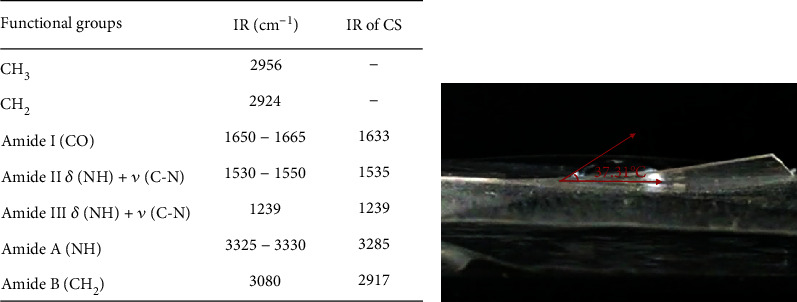
(a) Representing various functional group analyses in the AV/G scaffold. (b) The contact angles of the AV/G scaffold were found to be 37.31°. This implies that the hydrophilic characteristic has decreased with cross-linking.

**Figure 6:**
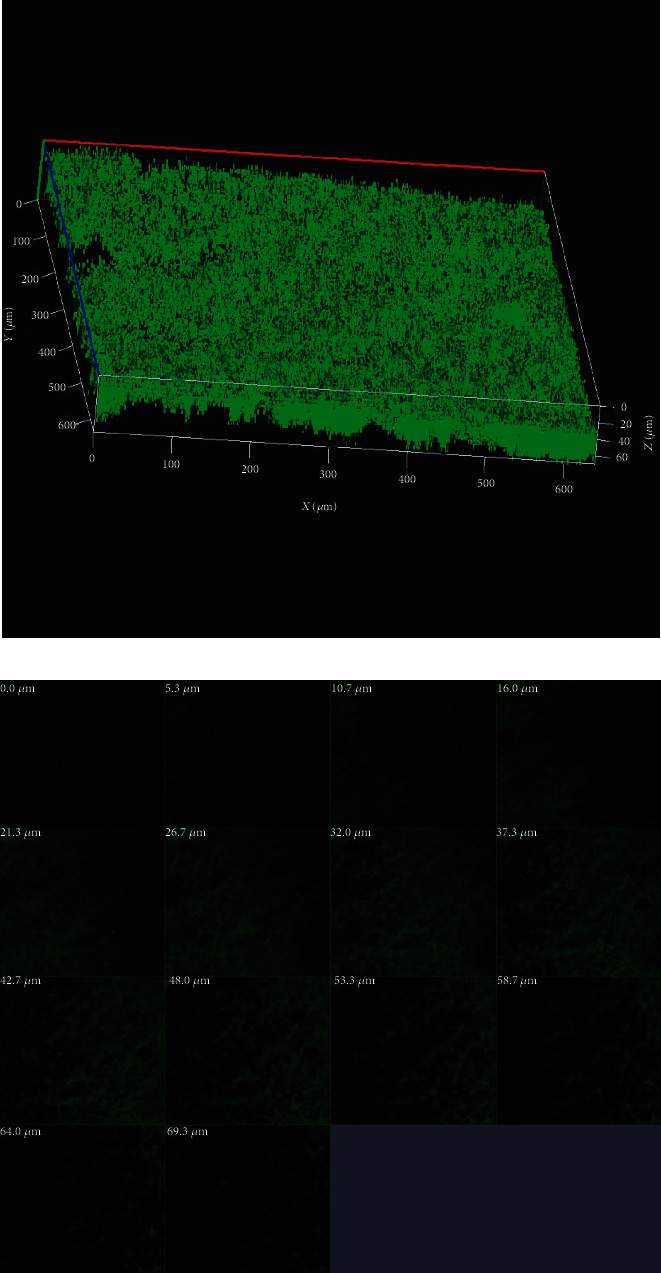
Confocal microscopic studies of the AV/G scaffold. (a) Shows the 3-dimensional view of the scaffold which measures the height of the scaffold to be 60 *μ*m. (b) Z-stack view of the scaffold where the black colour between green fluorescence represents pores.

Observations of DSC patterns reveal the characteristic endothermic peaks representing the temperature of dehydration (*T_D_*) and enthalpy of dehydration (*ΔH_D_*) of components of biomaterial.  Figure 3(b) shows the AV/G scaffold with *T_D_* value of 139.75°C and *ΔH_D_* of -26.40 W/g. CHNS was also estimated in (Figure 4(a)) and AV/G was found to have C% = 29.983, H% = 11.732, N% = 10.582, S% = 60.730, and O_2_% = 0. In Figure 4(b), graph represents X-ray diffraction of the AV/G scaffold. A huge amorphous peak was observed in the range of 2*θ* = 20°−40°, exhibiting compatibility.  Figure 5(a) shows the representation of functional groups CH_3_, CH_2_, Amide I (CO), Amide II *δ*(NH)+V(C-N), Amide III *δ*(NH) + V(C-N), Amide A (NH), and Amide B (CH_2_). The contact angle of *Aloe vera* with gelatin scaffold was found to be 37.31°, which shows wettability (Figure 5(b)), representing a good characteristic scaffold. The AV/G scaffold's tensile strength (MPa) was found to be 23.2, and an elongation break at 8.58 shows better tensile stress. Confocal images of the AV/G scaffold were observed due to the autofluorescence of *Aloe* *vera*. The 3D image and thickness of the scaffold were viewed under a confocal microscope (Figures 6(a) and 6(b)) at 40× magnification and were found to be about 60-80 *μ*m.

### 3.3. Biological Characterization of Scaffold

**Figure 7:**
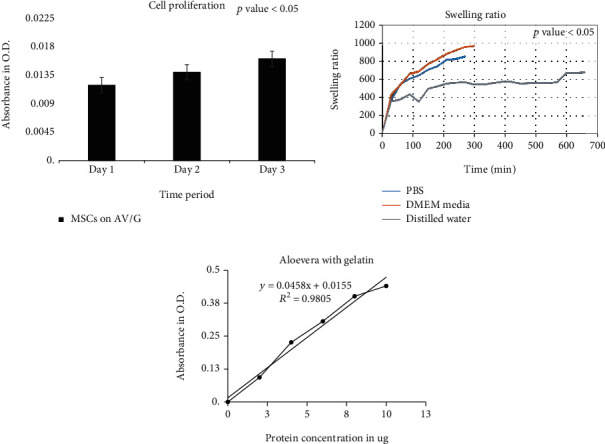
(a) The graph represents the AV/G scaffold biocompatibility study; analysis was carried out for viability and proliferation. An increase in the proliferation rate from day 1 to day 3 was observed using the MTT assay. (b) Shows the swelling ratio of AV/G scaffold compared with 1X PBS, DMEM media, and distilled water. (c) Shows protein estimation of the AV/G sample representing a bioactive scaffold.

**Figure 8:**
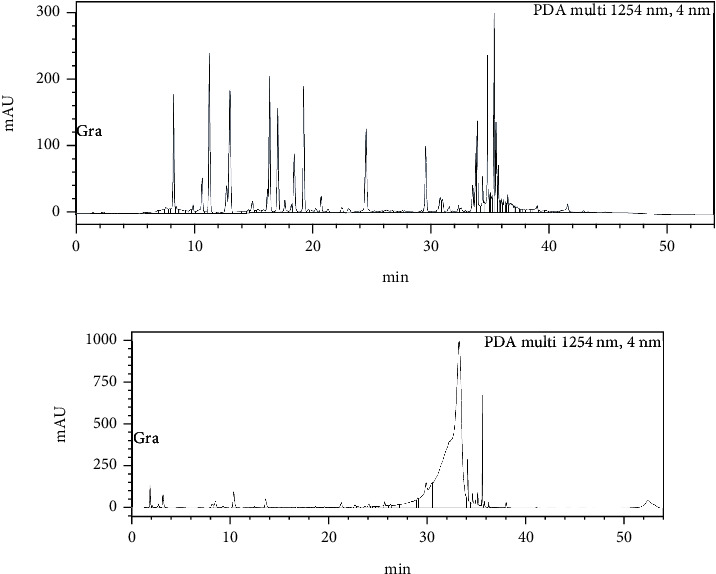
(a) Standard chromatogram of amino acids. (b) *Aloe vera* with 10% gelatin sample chromatogram.

**Figure 9:**
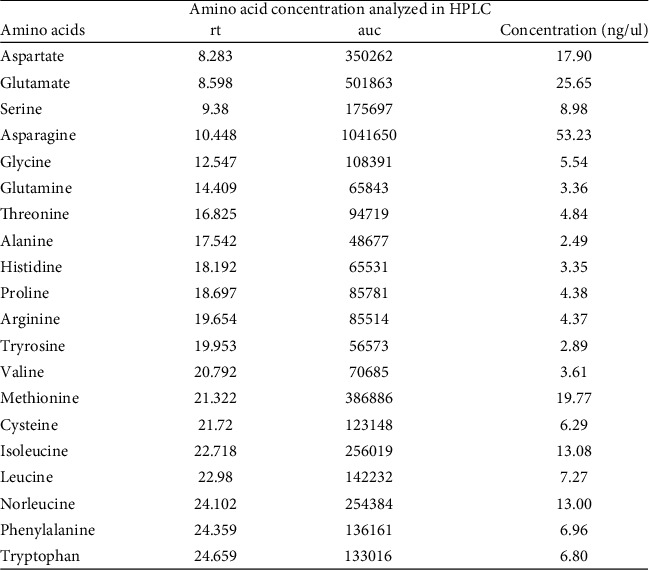
Amino acid concentration of AV/G analysed by HPLC.

**Figure 10:**
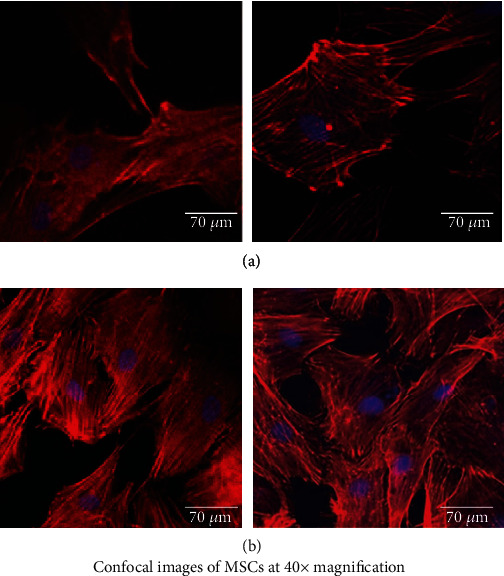
Confocal microscopic images at 40× magnification show human MSCs at P_5_ passage being cultured on an AV/G scaffold. (a) Shows the HG MSCs 24 hours after seeding onto the AV/G scaffold. F-actin-conjugated phalloidin stained in red colour. (b) Shows the HG MSCs 72 hours after seeding onto the AV/G scaffold. F-actin-conjugated phalloidin stained in red colour. Nucleus stained in DAPI blue colour.

**Figure 11:**
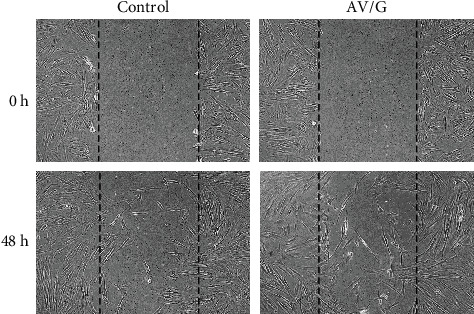
In vitro scratch wound healing assay. Human MSCs were injured, and cell migration assay with and without scaffold was viewed after 48 h. Images was obtained using an inverted phase contrast microscope at 10× magnification.

The biological characterization of the scaffold determines its compatibility and degradability. A MTT assay was performed to study (Figure 7(a)) the biocompatibility of AV/G and analyzed its viability and proliferation for 3 consecutive days. The proliferation rate of MSCs increases from days 1 to 3 which also shows the biocompatibility and viability of the scaffold. The swelling ratio of the freeze-dried AV/G scaffolds prepared showed a high degree of swelling (Figure 7(b)) upon placement in DMEM media at 37°C. It was also seen that the scaffold increases in ratio with 1X PBS, DMEM, and distilled water, respectively. The hydrophilicity of a scaffold is favorable for cell adhesion and growth. HPLC studies reveal amino acids in the protein content of 100 *μ*L of AV/G scaffold. This was deproteinized using 60% acetonitrile and PITC-derivatized for amino acid analysis using RP-HPLC. The AUC values (Figures 8(a) and 8(b)) of each analyte were converted to concentration (ng/*μ*L) using norleucine as a control. MSCs were cultured on the AV/G scaffold and treated with F-actin-conjugated phalloidin (red), and the nucleus was stained with DAPI (blue). HPLC results (Figure 9) showed amino acid concentrations in the *Aloe vera* with 10% gelatin gel.  Figure 10(a) shows MSCs at 24 hours, and Figure 10(b) shows MSCs at 72 hours that shows viability and proliferation. A wound healing assay was performed by creating a scratch, in which Figure 11 images show cell migration and gradual closure of the wound in MSCs with scaffold compared to control (MSCs without scaffold) was observed after 48 h at 10× magnification.

## 4. Discussion

Tissue engineering involves the principles of life sciences and engineering biomaterials for scaffold construction towards the development of biological substitutes which mimic, restore, maintain, and improve the function of an organ. It combines cells, materials, and appropriate biochemical elements to enhance or replace biological function. Fabrication of biologically active scaffolds has been generating promising results in tissue engineering and regenerative medicine. In 3D organ regeneration, a scaffold's topography and porosity are crucial factors [15]. In this study, human gingival MSCs were employed to design a tissue construct employing a three-dimensional AV/G scaffold for cellular adhesion, growth, and multiplication. Prior to this, *Aloe vera* gel with various concentrations of gelatin was lyophilized, and *Aloe vera* with 10% gelatin was optimized (Figure 1(a)). Lyophilization of *Aloe vera* with a gelatin (biopolymer) blend was studied for its functional benefits such as tensile properties, stability, porosity, and microstructural characteristics. Scanning electron microscopic (SEM) evaluation revealed that the AV/G scaffold was found to have a porous surface with random orientation. The SEM images of freeze-dried AV/G scaffold showed minimal porosity at 1500×, 4500×, and 5000× magnification (Figure 3(a)). These porous scaffolds have desirable characteristics for the purpose of cell integration which was similar to the one observed by Hassan and Peppas [16, 17]. The curve indicates a variation in the swelling of the scaffolds.

Mesenchymal stem cells were isolated from human gingival tissue gives similar yield to any adult tissue stem cells. Isolation of mesenchymal stem cells derived from human gingival tissue (Figure 1(b)) was viewed in phase contrast microscopy at 10× magnification (Figure 1(c)). MSCs were also characterized by differentiating them to osteocytes and adipocytes. Differentiated adipocyte and osteocyte from human gingival-derived mesenchymal stem cells were analyzed by alkaline phosphatase staining to determine osteocyte with the deposition of pink colour due to phosphate deposition in the cells and oil red O staining to determine the oil droplets in the adipocytes (Figures 1(d) and 1(e)). The images were viewed and imaged at 10× magnification under phase contrast microscopy. These cells were characterized in a flow cytometer, and 96.04% of cells were shown CD73 positive, 97.54% showed CD90 positive, and CD105 showed 96.77% of positive cells (Figure 2). Our results correlate with Zuk et al.'s [18] research, which described the separation and description of adipose-derived human stem cells. The differentiation of these cells into the adipogenic and osteogenic lineages was seen. The analysis of the cell surface markers CD73, CD90, and CD105 showed that the cell population expresses the known immnophenotype of MSC as reported by Lozito et al. [19]. The differentiation of MSCs into adipocyte (fat-containing cells) and osteocyte (bone cells) was analyzed by alkaline phosphatase staining that stains calcium and the phosphate deposited in the nucleus and oil red O staining that stains the oil drops inside the cells, respectively. So as to evaluate the property of MSCs, we characterized it with markers and subjected them to differentiation studies, and our result was similar to Vazin et al. [20].

Fourier transform infrared spectroscopy (Figure 5(b)) could be used as an effective method to define each component in a biomaterial containing *Aloe vera* and gelatin material. The position of the peaks, width, and intensity of the spectral band are sensitive to chemical conformational changes in molecules. The typical band reported in the literature assigned for gelatin is amide A(N-H stretching vibration) observed at around 3300 cm^−1^ which is similar to the results of the band absorbed by *Aloe vera* at 3314 cm^−1^ from the study conducted by Jithendran et al. [21]. Amide I was observed in the range of 1633–1639 cm^−1^, amide II was found at 1546–1551 cm^−1^, and amide III was in the range of 1239–1241 cm^−1^. Additional bands seen at 1450 cm^−1^ are attributed to identical -COO groups identified in the literature. The C-O stretching of *Aloe vera* polysaccharides is what causes the absorption peak to be noticed at 1079 cm^−1^. The carboxyl -COOH stretching bands are represented by the peaks in the spectra that are seen at approximately 1450 cm^−1^. Functional groups and FTIR were used to estimate CHNS (Figure 5(a)). The presence of a -helix with -turns and a distinctive absorption band indicating the peptide bonds (-CONH-) for amides I, II, and III are confirmed by observation of the spectral band. The N-H bonds' stretching vibration is what causes the peak of amide A. The stretching vibration of the C-O bonds is what gives amide I its peak. The amide II band is caused by N-H bending and C-H stretching vibration, and the amide III peak is caused by C-N stretching vibration. The spectral results exhibit aloe and gelatin's distinctive peaks, indicating that these ingredients were successfully incorporated into the scaffold's polymeric framework.

DSC exhibits small energy shifts that occur as matter transitions from a solid phase to a liquid crystalline phase and from a liquid crystal phase to an isotropic liquid phase. The DSC pattern (Figure 3(c)) depicts a shift in the energy of the AV/G scaffold on the basis of a *T_D_* value of 139.75°C and *ΔH_D_* -26.40 W/g.

In the intermolecular interaction in AV/G, AV also takes part. The distinctive endothermal peaks correspond to the temperature at which specific biomaterials dehydrate (TD) in a nitrogen-rich environment. The TD values and dehydration enthalpy (*H_D_*) are determined by DSC measurements. These findings imply that adding gelatin at a 10% weight-to-volume ratio to *Aloe vera* does not impact the pyrolytic property but stabilises the supercoil structure as seen by the higher solid-state *H_D_* values of the sample (lyophilized condition). The XRD graph was plotted with intensity along the *y*-axis and 2*θ* along the *x*-axis. XRD was used to analyse the composite scaffold's phase. The enormous amorphous peak seen in the region between 2*θ* = 20°−40° reduced the characteristic diffraction peaks for both AV/G (Figure 4(b)). The XRD data indicated that the gelatin and *Aloe vera* molecules in the scaffold had good compatibility and interaction. The broad peaks provided evidence that the synthesised scaffold was primarily amorphous. At the point where a liquid-vapor interface and a solid surface come together, the contact angle is a routinely measured angle through the liquid on the scaffold. The contact angle (Figure 5(b)) of *Aloe vera* with the gelatin scaffold was found to be 37.31°. It quantifies the wettability of a solid surface by a liquid through the Young equation. There is a specific equilibrium contact angle for every system comprising a given solid, liquid, and vapour at a given temperature and pressure. This implies that the hydrophilic characteristic has decreased with cross-linking. This may be due to the involvement of the chains in hydrogen bonding through hydroxyl groups.

The stress–strain relationship of a material is dependent on the flexibility of the polymer chains, and the strength of the material determines its tensile strength (MPa). The stress–strain curve for the prepared *Aloe vera* with gelatin scaffold's tensile strength (MPa) was found to be 23.2, and an elongation break at 8.58 shows a better tensile stress of the AV/G scaffold. Very high tensile strength may result in the scaffold staying with the wound bed longer until it regenerates, as reported by Suganya et al. [22]. AV/G scaffold being autofluorescent, the 3D image of the scaffold was viewed under a confocal microscope (Figures 6(a) and 6(b)), and the thickness of the scaffold was found to be about 60-80 *μ*m. However, the *Z*-stack of the AV/G scaffold shows minimal porosity. MSCs were cultured on the AV/G scaffold, and cell viability, compatibility, and proliferation were studied for 3 consecutive days through the MTT assay. For proliferative and viability studies, 1 × 10^4^ MSCs were seeded onto the AV/G scaffold. An increase in the proliferation rate from day 1 to day 3 was observed using the MTT assay (Figure 7(a)). The biocompatibility of AV/G was analyzed for viability and proliferation. The proliferation rate of MSCs increases from days 1 to 3 which also shows the biocompatibility and viability of the scaffold.

The swelling ratio of the freeze-dried *Aloe vera* with gelatin scaffolds prepared showed a high degree of swelling upon placement in water at 37°C. It was also seen that the scaffold increases in ratio with 1X PBS, DMEM, and distilled water, respectively (Figure 7(b)). It has been described that a higher degree of crosslinking results in lower water uptake, as proposed by Gupta et al. [23]. From the results, we can see that this point holds true for this study too. The swelling ratio aids in rigidity and polymer stability. The reduced swelling ratio could be attributed to a more rigid network formed by the interpolymer reactions, making the scaffold more stable. The scaffold's hydrophilicity promotes cell attachment and proliferation. Also, the water-holding ability would make it simpler for nutrients to diffuse from the scaffold to the cells in the culture system. In wound healing, this would balance the fluid loss from the body at the wound site [21]. Protein estimation (Figure 7(c)) of AV/G was done by the Bradford method which shows the presence of bioactive components in the AV/G scaffold and also an ECMmimicking niche.

HPLC results from Figures 8(a), 8(b), and 9 showed amino acid concentrations in the *Aloe vera* with 10% gelatin gel which were similar to the results of Hariharan et al. [14]. 100 *μ*L of AV/G was deproteinized using 60% acetonitrile and PITC-derivatized for amino acid analysis using RP-HPLC. The AUC values of each analyte were converted to concentration (ng/*μ*L) using norleucine as a control. The presence of amino acids shows the AV/G scaffold to be biologically active. For the adherence study, 10^4^ stem cells were seeded onto the AV/G scaffold. Images show that the lyophilized scaffold did not contain many pores, as seen using SEM analysis, and that both stem cells adhered to the surface. The cells were treated with F-actin-conjugated phalloidin (red), and the nucleus was stained with DAPI (blue). As *Aloe vera* autofluorescence green filter was muted to avoid background colouration. Images were taken after loading the cells onto the scaffolds. This was viewed at 40× magnification in confocal microscopy.  Figure 10(a) shows MSCs at 24 hours, and Figure 10(b) shows MSCs at 72 hours. The images of freeze-dried AV/G scaffold show pores and that the cells are embedded into the scaffold, and the MSCs are adhered and viable. Wound healing capacity of MSCs loaded AV/G in Figure 11. Images show strong wound-healing potential in terms of increasing MSC efficacy and wound closure in MSC-loaded AV/G scaffold to that of control (MSCs).

Regenerative medicine allows the human body to repair by producing new, useful tissue to replace damaged or missing ones. The endogenous system for repair and regeneration in the human body uses stem cells. Because stem cells serve as a backup in practically all of the essential organs, the idea of TERM is that the restoration of function of the stem cells is best accomplished in a bioscaffold. Regenerative medicine goes hand-in-hand with tissue engineering and stem cell technology. Tissue engineering has attracted much attention as a therapeutic tool which aids in providing 3-dimensional dynamic scaffold structure with biomaterial to mimic the lost organ. A cascade of signaling molecules is used in the dynamic and complex process of wound healing in order to rebuild tissue layers and cellular structures. *Aloe vera* gel has been investigated for decades and proven to be potentially useful in tissue matrix engineering. Ayurveda has established *Aloe vera* gel as a natural healing agent for ages. *Aloe vera* gel is used as a cosmetic and has been shown to hasten wound healing, protect, and soothe skin tissues. The anti-inflammatory property and increased collagen expression promote wound healing and antiaging. Stem cells are known to be unspecialized cells with high proliferative and differentiating properties. Stem cells are highly potent and relatively easy to isolate and expand with valuable properties for regenerative medicine. Signaling molecules, cell-to-cell contact, and interactions between stem cells and the extracellular matrix (ECM) around them make up a niche. Due to its distinct and appealing physicochemical and biological characteristics, *Aloe vera* has a bright future in tissue engineering applications. *Aloe vera* has been demonstrated to be helpful for tissue engineering as a material for scaffolds, drug-eluding implants, and wound dressings. Due to its characteristics, such as the effectiveness of bioactive compounds, biodegradability, biocompatibility, hydrophilicity, and synthesis of ECM, *Aloe vera*-based biomaterials have a new insight into the field of regenerative medicine. This study shows that an AV/G scaffold embedded with MSCs can be effectively used for wound healing and has been shown to be a promising candidate for tissue engineering applications.

## Data Availability

All of the data used to support the findings of this study are included within the article.
